# Increased Incidence and Altered Risk Demographics of Childhood Lead Poisoning: Predicting the Impacts of the CDC’s 5 µg/dL Reference Value in Massachusetts (USA)

**DOI:** 10.3390/ijerph9113934

**Published:** 2012-10-30

**Authors:** Phoebe Handler, Daniel Brabander

**Affiliations:** 1 Wellesley College, Department of Environmental Studies, 21 Wellesley College Road, c/o Daniel Brabander, Wellesley, MA 02481, USA; 2 Geosciences Department, Wellesley College, 21 Wellesley College Road, Wellesley, MA 02481, USA; Email: dbraband@wellesley.edu; 3 Exposure, Epidemiology and Risk Program, Harvard School of Public Health Landmark Center, Room 404 West, 401 Park Drive, Boston, MA 02215, USA

**Keywords:** lead poisoning, community health, risk, demographics, public health, Massachusetts, CDC

## Abstract

In May 2012, the CDC adopted a new sliding scale reference value for childhood lead poisoning, reducing the former 10 µg/dL benchmark by half. Using Massachusetts (MA) as a model state, we estimated the change in the population of 9–47 month-olds at risk for lead poisoning. We then examined the impact of the 5 µg/dL reference value on the demographic characteristics of lead risk in MA communities. We find that the new CDC benchmark will lead to a 1470% increase in childhood lead poisoning cases among 9–47 month-olds in MA, with nearly 50% of the examined communities experiencing an increased prevalence of lead poisoning. Further, the top 10 MA communities with BLLs ≥5 µg/dL have significantly fewer foreign-born residents and significantly larger white populations than the highest risk communities formerly identified by the MA Childhood Lead Poisoning Prevention Program. The CDC’s new 5 µg/dL lead poisoning benchmark will drastically increase the number of children with elevated BLLs and alter the distribution and demographics high-risk communities in MA.

## 1. Introduction

At the beginning of 2012, childhood lead poisoning was not considered a major public health issue in the United States; in fact, the reduction in lead poisoning incidence was extolled by the Centers for Disease Control (CDC) as one of the “Great Public Health Achievements” of the United States from 2001–2010 [1]. With a blood lead level (BLL) benchmark of 10 µg/dL, less than 1% of our nation’s 1 to 5 year old children were considered to have lead poisoning.

In May 2012, the CDC adopted a new protocol for establishing elevated BLLs. The 97.5th percentile BLL of U.S. children aged 1 to 5 years will now be used as a sliding reference value for lead poisoning in the United States. The CDC will update this number every four years using the most current childhood BLL distribution reported by the National Health and Nutrition Examination Survey [2]. Currently, the U.S. lead poisoning reference value is 5 µg/dL, half of the former 10 µg/dL benchmark.

This new reference value is a timely response to a growing scientific consensus that permanent damage from lead exposure occurs at BLLs below 10 µg/dL [2,3,4,5,6,7,8,9,10]. In fact, the CDC has officially changed the language it uses to talk about lead poisoning to acknowledge that there is no safe level of lead exposure [2].

The CDC’s benchmark reduction will surely increase the number of children in the U.S. who are considered to have lead poisoning. This study aims to quantify the demographics of the expanded cohort of at-risk children for lead poisoning using the state of Massachusetts as a model.

Further, an expansion in the pool of children at risk for lead poisoning may alter the traditional demographic risk factors associated with the ailment. The body of literature exploring and defining the population risk demographics of childhood lead poisoning in the United States is well established [5,11,12,13,14]. A second aim of this study is to examine how the reduced lead poisoning benchmark will alter the demographic correlates of lead poisoning in Massachusetts.

Proposed estimates of the total number of children newly considered to have lead poisoning [15] do not take into account the inequitable distribution of at-risk children across the nation [16,17]. Therefore, localized models of lead poisoning risk offer an important tool for identifying community-scale risk patterns that are not discernable from models with larger geographic scopes. In order to create a state-scale model for a population-based lead risk assessment, Massachusetts was selected for its heterogeneous population and accessible state-reported BLL measures.

The new lead poisoning reference value will place pressure on public health departments and organizations across the nation. In order to address the growing cohort of high-risk children, there is a need for inexpensive and effective methods of identifying high-risk populations who merit focused primary prevention efforts. By exploring BLL patterns and demographic risk profiles for lead exposure in MA, this study identifies high-risk communities that particularly merit lead poisoning prevention interventions. We estimate the increase in lead-poisoned children in MA under the new CDC reference value and evaluate the impact of this expanded risk cohort on the demographic predictors of childhood lead poisoning in MA communities.

## 2. Experimental Section

The frequencies of BLLs for children aged 9 to 47 months, the age group used for childhood lead poisoning analysis by the MA Department of Public Health, in MA towns and cities (n = 349) were gathered from a publicly available online database provided by the Massachusetts Department of Public Health’s Bureau of Environmental Health [18]. BLLs, in the intervals of 0–9 µg/dL, 10–14 µg/dL, 15–24 µg/dL and *≥*25 µg/dL, were collected on the community scale for children aged 9–47 months from the year 2007. MA cities with incomplete or withheld BLL data were removed from the list.

Though it was not available online, the MA Department of Public Health issued the 0–10 µg/dL BLL distributions for children aged 9–47 months in 2007 by request at no cost [19]. These data were binned for 0 µg/dL, 1–4 µg/dL and 5–10 µg/dL as percentages of the sampled population with BLLs from 0–10 µg/dL.

Demographic variables relevant to lead poisoning risk, including median household income, poverty rate, homeownership rate, percent foreign-born, and educational attainment, were collected from the U.S. Census for Massachusetts from data collected over the period of 2006–2010 [20]. U.S. Census data from the 2010 Census Interactive Population Map was then used to gather race/ethnicity and demographic information for each MA community [21]. Communities with incomplete census profiles were removed from the list of eligible locations.

Correlation analyses were completed using Microsoft Excel (Microsoft Corporation, Redmond, WA, USA, 2004). Pearson correlation and two-tailed significance values were calculated using SPSS predictive analytic software version 19 (IBM, Armonk, NY, USA).

## 3. Results and Discussion

### 3.1. MA Communities

Fifty-four communities had complete BLL, race/ethnicity, and demographic profiles. The total population of these communities accounts for approximately 50% of the state population (3,139,064 persons). In the 54 MA communities, 79.7% of the population is white, 12.4% is Hispanic, 5.5% is black, 4.9% is Asian, and 16.2% is foreign-born. The average median household income is $61,976, 11.7% of the population falls below the federal poverty level, and homeownership rate is 59.1%.Of those aged 25 and above, 86.5% graduated from high school and 35.2% have a Bachelor’s or advanced degree.

### 3.2. Estimating the Expanded Pool of at-Risk Children

**Figure 1:**
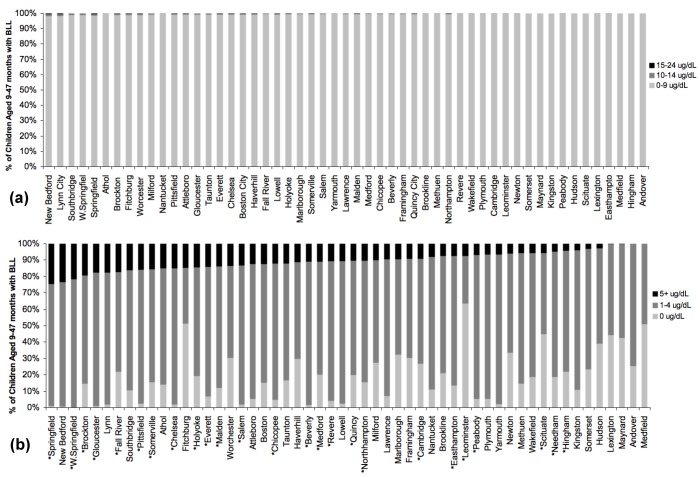
Distribution of BLLs by MA community for children aged 9–47 months in 2007.** (a)** Distribution of BLLs binned according to the old CDC lead reference level of 10 µg/dL. **(b)** Distribution of BLLs binned according to the new 5 µg/dL CDC reference value. Locations marked with an asterisk (*) have increased risk ranking under the new reference value.

In order to assess changes in childhood lead poisoning risk under the CDC’s new 5 µg/dL reference value, we ranked the 54 communities in a series of binning schemes. First, to represent lead risk under the former 10 µg/dL benchmark, the BLLs of MA children were divided into bins of 0–9 µg/dL, 10–14 µg/dL, and 15–24 µg/dL, as displayed in Figure 1(a). Only 0.7% of 1 to 5 year old children in MA had BLLs of 10 µg/dL or higher in 2007, a very small minority. To identify children formerly masked by the 10 µg/dL reference value, the 9–47 month-old MA population with BLLs from 0–10 µg/dL in 2007 was divided into bins of 0 µg/dL, 1–4 µg/dL and 5–10 µg/dL. Of the 99.3% of children with BLLs from 0–10 µg/dL, 10.5% of children had BLLs in the 5–10 µg/dL range. The distribution of lead poisoning risk under the CDC’s new 5 µg/dL reference value is shown in Figure 1(b), which presents the BLLs of 9–47 month-olds in bins of 0 µg/dL, 1–4 µg/dL, and ≥5 µg/dL.

In Massachusetts, the new 5 µg/dL reference value will increase the number of children considered to have lead poisoning by 1470%. Nearly half of the examined communities will experience increased lead poisoning prevalence under the new reference value.

The ten MA communities with the highest proportion of children aged 9–47 months with BLLs ≥5 µg/dL are displayed in Table 1. Of the top ten MA communities for BLLs from ≥5 µg/dL, 40% were not included in the Massachusetts Childhood Lead Poisoning Prevention Program’s “High Risk Communities for Childhood Lead Poisoning” list, which singled out the cities in MA where lead poisoning was a large threat from 2002 to 2007 [22] (Table 1). Further, all of the top risk communities for BLLs ≥5 µg/dL changed in their ranking when compared to the distribution communities under the 10 µg/dL benchmark displayed in Figure 1(a) (see Table 1).

**Table 1:** Ten MA communities with the highest proportion of children aged 9–47 months with BLLs from ≥5 µg/dL in 2007, ranked by percent of population with BLLs ≥5 µg/dL, including the cities’ rankings under the 10 µg/dL benchmark and inclusion on the MA CLPPP high-risk list.

BLL ≥5 µg/dL ranking	City	% of sampled population with BLL ≥5 µg/dL	BLL ≥10 µg/dL ranking	Inclusion on MA CLPPP High Risk List (2002-2007)
1	Springfield	25.0%	5	√
2	New Bedford	23.7%	1	√
3	West Springfield	22.2%	4	×
4	Brockton	19.7%	7	√
5	Gloucester	18.0%	14	×
6	Lynn	18.0%	2	√
7	Fall River	17.4%	20	√
8	Southbridge	16.4%	3	×
9	Pittsfield	16.1%	12	×
10	Somerville	16.0%	24	√

For the top ten highest risk communities under the new CDC reference value, the proportion of young children with BLLs ≥5 µg/dL ranges from 16% to 25%. These communities’ risk rankings are strikingly different than they were under the 10 µg/dL binning scheme, with changes in rank ranging from 1 to 14. The MA CLPPP’s High Risk Communities List has become an outdated measure of lead poisoning risk.

### 3.3. Evolving Lead Poisoning Risk Demographics

For the new top ten MA communities at risk for childhood lead poisoning, 74.3% of the population is white, 15.9% is Hispanic, 9.5% is black, 3.2% is Asian, and 16.5% is foreign-born. The average median household income is $46,216, 16.8% of the population falls below the federal poverty level, and the homeownership rate is 50.7%. Of the population above age 25, 80.5% graduated from high school and 23.3% have a Bachelor’s or advanced degree.

Correlation analysis was completed for all 54 MA communities between community BLLs ≥5 µg/dL and the primary demographic risk factors for lead poisoning risk, including median home income, poverty rate, educational attainment, homeownership rate, and race/ethnicity. The results of the correlation analyses are offered in Table 2.

The demographic factors traditionally associated with lead poisoning risk in the United States were strongly correlated with BLLs ≥5 µg/dL in 9–47 month-old MA children. Our analysis demonstrated that income level and poverty rate have the strongest correlations with BLLs in MA (*p* < 0.001, r^2^ = 0.4). Socio-economic standing, therefore, is the strongest demographic correlate to lead poisoning rates in Massachusetts.

**Table 2:** Demographic variables and BLL correlation measures for correlation of BLLs ≥5 µg/dL with demographic variables for all MA towns.

Variable	R^2^ value	*p* value
Median Household Income	0.4	<0.001
Poverty Rate	0.4	<0.001
Educational Attainment	0.3	<0.001
African American Population	0.3	<0.001
Homeownership Rate	0.3	<0.001
Hispanic Population	0.3	0.01
White Population	0.2	<0.005

To assess the impacts of the expanded risk population for childhood lead poisoning on the demographic profiles traditionally associated with the ailment, we compared the average demographic risk factor values for the top 10 communities for BLLS ≥5 µg/dL with the communities on the MA CLPPP’s high-risk list for the years 2002 to 2007 [22] (see Table 3).

**Table 3:** Comparison of 2007 average lead poisoning risk demographic measures from the top 10 MA communities with BLLs ≥5 µg/dL and the MA CLPPP’s highest risk communities.

Variables	Top 10 MA Communities with BLLs ≥5 µg/dL in 2007	MA CLPPP High Risk Communities List 2002-2007	*p* value
% Foreign-Born	16.5	25.3	0.03 *
% White	74.3	60.5	0.05 *
Homeownership Rate	50.7	42.9	0.08
% Below Poverty Level	16.8	20.6	0.10
% Hispanic	15.9	27.9	0.15
% High School Graduates	80.5	75.9	0.19
Median Household Income	$46,216	$43,446	0.51
% African American	9.5	12.9	0.42
% with Bachelor’s Degree	23.3	23.0	0.86

* Significant to the *p* ≤ 0.05 level

For the foreign-born (*p* = 0.03) and white population (*p* = 0.05) proportions, there was a significant difference between the old CLPPP top risk communities and the new top ten risk communities under the 5 µg/dL reference value. The percent of 9–47 month-old children with BLLs ≥5 µg/dL was about 19.2%, marking a 22.3% increase over that same population proportion for the MA CLPPP’s highest risk communities (15.7%). The expanded pool of children with BLLs above the national benchmark will alter the extent of risk demographics for lead poisoning in MA. The high-risk communities newly identified by the 5 µg/dL benchmark have distinctly different social, economic, and demographic profiles than those with the highest risk under the old lead poisoning benchmark.

## 4. Conclusions

This study finds that the MA lead poisoning rate is 15.7 times greater under the 5 µg/dL reference value than it was under the former 10 µg/dL reference value. Further, the new reference value will distinctly alter the landscape of lead risk in MA, with nearly 50% of the examined communities experiencing an increased risk for lead poisoning.

The demographic characteristics associated with lead poisoning risk have shifted under the new benchmark. Many of the risk conditions traditionally related to lead poisoning are significantly different in the new high-risk MA communities, compared with the high-risk communities previously identified by the MA CLPPP. Specifically, the top ten MA communities with BLLs ≥5 µg/dL have significantly fewer foreign-born residents and significantly larger white populations than the former highest risk communities.

The expanded pool of lead poisoned children and the shifting community demographics of lead poisoning suggest a need for increased and expanded medical and public health attention to local communities in MA. Not only are there more children who merit primary prevention outreach, many of the affected communities are not the “usual subjects” of lead poisoning interventions.

In understanding the impacts of the new CDC reference value on lead poisoning risk in MA communities, prevention efforts must address the changing landscape of lead risk. To more accurately and efficiently identify this expanded risk cohort, practitioners will need to assess risk on the local level rather than relying on national data. The low-cost and accessible methods used in this analysis may be used as a model for local and regional public health organizations that wish to efficiently and effectively identify communities with high-risk populations for lead poisoning.

Lead poisoning remains a developmental threat to children in the United States. As the CDC’s policy catches up to the scientific consensus that there is no safe level of lead exposure, we must now acknowledge that childhood lead poisoning is not yet the “Great Public Health Achievement” [1] it was so recently thought to be. Now more than ever, lead poisoning prevention efforts should focus on those most at risk for exposure.

The CDC’s new reference value will increase the number of children considered to have lead poisoning by 1470% and will markedly change the community-scale demographics of lead poisoning risk in Massachusetts. The impacts of the new 5 µg/dL benchmark should be examined on localized scales across the nation to more accurately identify and address regional lead poisoning prevalence.
